# Engineering CAR-T cells for solid tumors: Overcoming the microenvironment through integrated design and clinical translation

**DOI:** 10.18632/oncoscience.666

**Published:** 2026-07-29

**Authors:** Samuel Obiosa Onyekweli, Gloria Osayamen Omoruyi, Christopher Oloruntoba Akintayo, Samuel Kehinde Olaniyi

**Affiliations:** ^1^Department of Internal Medicine, Obafemi Awolowo University Teaching Hospital Complex, Ile-Ife, Nigeria; ^2^Department of Physiology, Afe Babalola University, Ado-Ekiti, Nigeria

**Keywords:** CAR-T cell therapy, solid tumors, tumor microenvironment, immunotherapy engineering, clinical translation

## Abstract

Chimeric antigen receptor T-cell (CAR-T) therapy has produced remarkable therapeutic results in blood cancers, while its application to solid malignancies remains limited by a pooled objective response rate of approximately 9%. This gap stems from core biological obstacles: heterogeneous antigen expression, physical inaccessibility within dense stromal architectures, and immunosuppressive microenvironments that drive T-cell exhaustion through epigenetically fixed transcriptional programs. The period spanning 2024–2025 represents a pivotal turning point. GD2-targeting CAR-T cells delivered intracerebroventricularly achieved durable complete responses (including one sustained beyond 30 months) in H3K27M-mutated diffuse midline gliomas. CLDN18.2-targeting satricabtagene autoleucel demonstrated randomized superiority over physician’s choice in advanced gastric cancer (progression-free survival HR 0.37). GPC3-targeting CAR-T cells armored with a dominant-negative TGF-β receptor achieved objective response rates of 50–57% in hepatocellular carcinoma, representing a three- to four-fold improvement over unarmored predecessors. These breakthroughs reflect a paradigm shift from potency-driven engineering toward resilience-based design: metabolic armoring via autocrine IL-10 and IL-15, epigenetic protection through DNMT3A disruption and c-Jun overexpression, logic-gated targeting via synNotch circuits, and microenvironmental shielding through dominant-negative receptors. Beyond the local microenvironment, emerging recognition of systemic neuroendocrine-immune dysregulation further informs CAR-T persistence and fitness considerations. This review synthesizes the mechanistic insights, engineering strategies, clinical evidence, and emerging platforms, including *in vivo* lentiviral CAR-T generation, that define the current landscape, and proposes a tiered framework for next-generation solid tumor CAR-T development, while explicitly acknowledging the limitations and unknowns that persist.

## INTRODUCTION

The clinical trajectory of chimeric antigen receptor T-cell (CAR-T) therapy exemplifies both the revolutionary promise and ongoing challenges of engineered cellular immunotherapy. Pediatric patients with relapsed B-cell acute lymphoblastic leukemia (B-ALL) attain complete remission rates surpassing 80% following CD19-directed tisagenlecleucel [1], while adults with refractory diffuse large B-cell lymphoma (DLBCL) achieve durable responses with axicabtagene ciloleucel [2]. This therapeutic approach has subsequently extended to multiple myeloma, where B-cell maturation antigen (BCMA)-directed constructs (idecabtagene vicleucel and ciltacabtagene autoleucel) have yielded exceptional response rates of 73% and 98%, respectively, in heavily pretreated patients [3, 4].

In contrast, solid tumor outcomes have been characterized by markedly different statistics. A 2019 meta-analysis of 22 studies encompassing 262 patients estimated the pooled objective response rate (ORR) for CAR-T therapy in solid malignancies at 9% (95% CI 4–16%) [5]. This figure, nearly an order of magnitude below hematologic benchmarks, provided a stark statistical benchmark, quantifying the so-called “solid tumor barrier.” The biological foundations underlying this shortfall are multifaceted: physical exclusion of T cells by desmoplastic stroma, metabolic starvation within the tumor microenvironment (TME), antigen heterogeneity permitting immune escape, and an epigenetically encoded exhaustion program that renders infiltrating T cells functionally inert [6, 7].

However, the period spanning 2024–2025 has seen a critical turning point. Three groundbreaking programs illustrate the shift from insurmountable failure to tractable engineering problem.

### Diffuse midline glioma (DMG)

The GD2-targeting CAR-T program reported by Monje, Mahdi, Majzner, and colleagues demonstrated that intracerebroventricular delivery achieves durable complete responses in H3K27M-mutated tumors, including one sustained beyond 30 months [8]. This was preceded by the initial 4-patient proof-of-concept report [9] and constitutes an unprecedented outcome for a disease with median survival below one year.

### Gastric cancer

CLDN18.2-targeting satricabtagene autoleucel (satri-cel) provided, in the CT041-ST-01 Phase 2 randomized controlled trial, the first evidence of CAR-T superiority over standard-of-care in a solid malignancy, with a progression-free survival (PFS) hazard ratio of 0.37 (*p* < 0.0001) and an overall survival (OS) hazard ratio of 0.69 (*p* = 0.04) [10, 11].

### Hepatocellular carcinoma (HCC)

The GPC3-targeting C-CAR031, incorporating a dominant-negative TGF-β receptor type II (dnTGF-βRII), achieved an ORR of 50–57% and a disease control rate exceeding 90% in advanced HCC [12], representing a three- to four-fold improvement over unarmored predecessors [13].

This review offers a thorough synthesis of these breakthroughs. We analyze the conceptual transition from “potency engineering” (characterized by stronger costimulatory domains and pro-inflammatory cytokines) to “resilience engineering,” defined by metabolic armoring, epigenetic protection, and microenvironmental shielding. We explore how localized delivery approaches have transformed protected niches into treatment-accessible zones, how synthetic biology circuits enable precision targeting, and how the gut microbiome has surfaced as an unexpected factor influencing results. We further extend the conceptual scope to include systemic neuroendocrine-immune regulation, which has emerged as an additional dimension shaping CAR-T persistence and fitness. Finally, we present a stratified evidence-driven framework for rational next-generation solid tumor CAR-T development.

## THE SOLID TUMOR BARRIER: DECONSTRUCTING THE FAILURE

**Figure 1:**
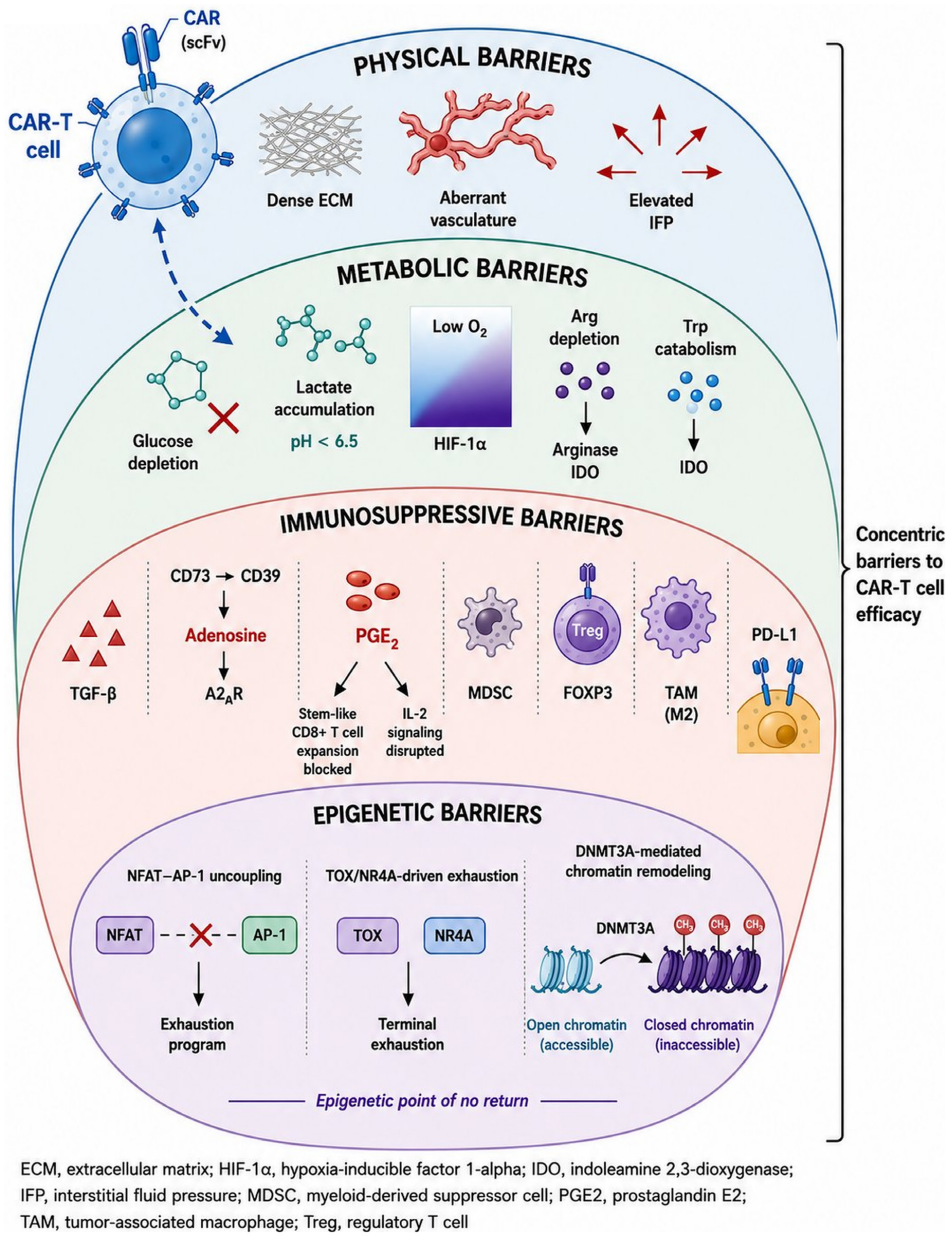
The solid tumor barrier: A multi-layered defense. Schematic representation of the concentric barriers confronting CAR-T cells in solid tumors. Physical barriers include dense extracellular matrix, aberrant vasculature, and elevated interstitial fluid pressure. Metabolic barriers encompass glucose depletion, lactate accumulation, hypoxia, and arginine/tryptophan catabolism. Immunosuppressive barriers include TGF-β, PGE2, adenosine (CD73/CD39/A2AR axis), MDSCs, Tregs, and tumor-associated macrophages. Epigenetic barriers involve NFAT–AP-1 uncoupling, TOX/NR4A-driven exhaustion programming, and DNMT3A-mediated chromatin remodeling. Abbreviations: ECM, extracellular matrix; HIF-1α, hypoxia-inducible factor 1-alpha; IDO, indoleamine 2,3-dioxygenase; IFP, interstitial fluid pressure; MDSC, myeloid-derived suppressor cell; PGE2, prostaglandin E2; TAM, tumor-associated macrophage; Treg, regulatory T cell.

To grasp the significance of these recent advances, it is essential to first dissect the biological defenses that have been overcome. The “solid tumor barrier” comprises not one barrier but multiple layered defensive mechanisms that have traditionally overwhelmed conventional second-generation CAR-T cells [6] (Figure 1).

### The efficacy gap

The efficacy gap between hematologic and solid tumor CAR-T can be best understood via effector-to-target (E:T) ratios and microenvironmental accessibility. In leukemia, infused CAR-T cells enter the bloodstream and immediately encounter their targets. The compartment is fluid, permitting high mixing rates and exponential expansion. Solid tumors, conversely, function as organ-like entities marked by aberrant vasculature, elevated interstitial fluid pressure (IFP), and dense extracellular matrix (ECM) composed of collagen and hyaluronan [6]. A systemically infused CAR-T cell must extravasate against a pressure gradient, penetrate the stromal barrier, and survive in a hypoxic tumor core, constituting a delivery problem of the first order.

### Tonic signaling and the seeds of exhaustion

Prior to TME exposure, CAR-T cells may harbor intrinsic susceptibility to dysfunction. Long and colleagues demonstrated that antigen-independent clustering of certain scFv domains (particularly GD2-specific 14g2a) induces tonic CD3-ζ phosphorylation, constitutively activating downstream signaling in the absence of antigen [14]. This constitutive signaling promotes gradual T-cell exhaustion and functional decline. Importantly, the selection of costimulatory domain influences this process: 4-1BB-containing CARs ameliorate tonic signaling-induced exhaustion by augmenting mitochondrial biogenesis, whereas CD28-containing CARs augment exhaustion by driving glycolytic metabolism and effector differentiation [14, 15]. Kawalekar and colleagues extended this observation, demonstrating that 4-1BB costimulation promotes central memory differentiation and sustained oxidative phosphorylation, while CD28 costimulation favors effector memory and glycolysis [15]. This difference carries significant consequences for solid tumor applications, where T-cell persistence, rather than peak expansion, is the rate-limiting determinant of efficacy.

### The epigenetic “point of no return”

Arguably the most significant conceptual advance in the past five years has been reframing T-cell exhaustion from a reversible functional condition to an epigenetically locked transcriptional state.

#### The NFAT–AP-1 uncoupling

Under normal physiological circumstances, T-cell receptor stimulation drives nuclear translocation of NFAT, which partners with AP-1 (Fos/Jun heterodimers) to activate effector gene transcription. In the solid TME, chronic antigen stimulation combined with hypoxia leads to progressive downregulation of AP-1 components. This generates “partnerless” NFAT, which actively redirects transcription toward exhaustion-promoting loci, activating expression of TOX and NR4A family members [16, 17]. Seo and colleagues further demonstrated that TOX and TOX2 cooperate with NR4A transcription factors to impose the exhaustion program [18].

#### The chromatin scar

TOX and NR4A recruit epigenetic modifiers, most critically the de novo DNA methyltransferase DNMT3A. Weber and colleagues quantified this transformation using ATAC-seq: approximately 48,000 chromatin accessibility peaks change within the first seven days of continuous tonic signaling [19]. This massive epigenomic restructuring physically closes enhancer regions required for effector cytokine production. Prinzing and colleagues subsequently demonstrated that CRISPR-mediated knockout of DNMT3A prevents this exhaustion program, maintaining CAR-T proliferative capacity through an IL-10-dependent mechanism [20].

#### The checkpoint blockade fallacy

This epigenetic understanding clarifies why pairing CAR-T cells with PD-1 checkpoint inhibitors has repeatedly failed to rescue efficacy in solid tumors. Anti-PD-1 antibodies release a cell-surface signaling brake, but if the downstream chromatin machinery has been epigenetically locked, releasing the brake achieves nothing. The cell cannot transcribe effector genes even when the inhibitory signal is removed. The deficiency originates in the nucleus rather than at the membrane.

### The metabolic desert

In addition to epigenetic reprogramming, the TME exerts substantial metabolic pressure. The Warburg effect depletes local glucose to levels insufficient for the glycolytic burst required for T-cell cytotoxicity. Concurrently, hypoxia stabilizes HIF-1α in tumor cells, driving VEGF and PD-L1 expression while directly inhibiting T-cell proliferation. Lactate accumulation acidifies the microenvironment (often to pH < 6.5), directly compromising cytolytic activity [6]. Recent metabolic engineering approaches, including acetate supplementation to rescue effector function via ACSS-dependent histone acetylation [21] and L-arginine supplementation to shift T-cell metabolism toward oxidative phosphorylation and central memory phenotypes [22], have shown that these metabolic obstacles can be addressed through rational intervention. Fultang and colleagues took this further, engineering CAR-T cells to express arginine resynthesis enzymes (ASS and OTC), enabling proliferation in arginine-depleted microenvironments [23].

### Prostaglandin E2: A lipid mediator of immune suppression

Beyond cytokine- and metabolite-mediated suppression, prostaglandin E2 (PGE2) has emerged as a pivotal lipid mediator of TME immunosuppression. Tumor cells and myeloid populations within the TME secrete PGE2 abundantly, where it acts on EP2/EP4 receptors expressed on T cells. Two complementary 2024 studies established the mechanistic centrality of this pathway. Lacher and colleagues demonstrated that PGE2 specifically restricts the effector expansion of tumour-infiltrating stem-like CD8+ T cells, thereby limiting the reservoir of self-renewing T cells available for sustained antitumor activity [24]. Morotti and colleagues, in a companion study, showed that PGE2 disrupts IL-2 signalling and mitochondrial function in tumour-infiltrating lymphocytes, compromising both expansion and effector capacity [25]. These findings position PGE2 alongside TGF-β and adenosine as a major axis of immune suppression with direct implications for CAR-T cell function, and motivate consideration of cyclooxygenase inhibitors or EP receptor antagonists as combination strategies. The integrated solid tumor barrier, including this lipid-mediated dimension, is depicted in Figure 1.

## ENGINEERING STRATEGIES: THE PARADIGM SHIFT TO RESILIENCE

The development of CAR-T engineering can be viewed as a shift between two philosophical phases. The “Potency Era” (approximately 2010–2020) was characterized by pressing the accelerator by adding stronger costimulatory domains and pro-inflammatory cytokines. The current “Resilience Era” (2021–present) has reversed this approach. Instead of maximizing activation, contemporary engineering focuses on shielding the T cell from hostile environmental conditions [7, 26].

### c-Jun overexpression: Restoring the AP-1 partnership

The discovery of the NFAT–AP-1 uncoupling mechanism directly motivated a compensatory engineering strategy. Lynn and colleagues demonstrated that constitutive overexpression of c-Jun in CAR-T cells restores the AP-1 component of the NFAT partnership, preventing the transcriptional redirection toward exhaustion loci [27]. c-Jun-overexpressing CAR-T cells exhibited enhanced expansion, increased IL-2 production, resistance to exhaustion, and improved anti-tumor activity in multiple solid tumor models. This discovery was notable for its straightforward elegance: rather than blocking the exhaustion pathway downstream, c-Jun overexpression addresses the initiating event, specifically the absence of a NFAT binding partner. The c-Jun-modified platform is being advanced clinically by Lyell Immunopharma, and Zuo and colleagues reported the first Phase I trial using c-Jun-modified CARs in acute myeloid leukemia [28], though solid tumor clinical data remain pending.

### Metabolic armoring: The IL-10 paradigm shift

IL-10 has been classified for decades as a prototypical immunosuppressive cytokine. The resolution of this apparent paradox lies in the distinction between paracrine and autocrine signaling. Zhao and colleagues demonstrated that CAR-T cells engineered to constitutively secrete IL-10 achieved complete regression of established solid tumors where standard CARs uniformly failed [29]. Autocrine IL-10 signaling promotes mitochondrial fusion, cristae integrity, and oxidative phosphorylation via the mitochondrial pyruvate carrier (MPC). Critically, IL-10 armoring also upregulated AP-1 transcription factors (Jun, Fos), directly counteracting the partnerless NFAT mechanism. Thus, IL-10 armoring concurrently targets both the metabolic and epigenetic aspects of exhaustion, representing a dual mechanism that may explain its preclinical efficacy.

### Cytokine armoring: IL-15, IL-18, and IL-21

Beyond IL-10, cytokine armoring has been explored with IL-15, IL-18, and IL-21. Steffin and colleagues reported clinical data on IL-15-armored GPC3-targeting CAR-T cells in patients with solid cancers, achieving a 66% disease control rate in the optimized cohort, representing the strongest clinical support for cytokine-armored CARs to date [30]. The mechanism of IL-15 armoring differs fundamentally from IL-10: IL-15 preserves stem cell memory (T_SCM) phenotype by reducing mTORC1 activity [31].

IL-18 armoring represents a complementary strategy with distinct mechanistic logic. Chmielewski and Abken demonstrated that CAR-T cells engineered to release IL-18 convert to a T-Bet^high^/FoxO1^low^ effector phenotype with augmented activity against advanced solid tumors, including independence from host immunity [32]. Hu and colleagues confirmed this approach across human and murine CAR-T constructs, showing IL-18 secretion enhances antitumor immunity in both syngeneic and xenograft models [33]. Avanzi and colleagues extended this paradigm by demonstrating that IL-18-secreting CAR-T cells engage both direct cytotoxicity and activation of the endogenous immune system, broadening antitumor reach beyond the engineered population [34]. Together, these approaches illustrate how cytokine armoring can be tuned not only for T-cell-intrinsic fitness (IL-10, IL-15) but also for orchestration of the broader immune microenvironment (IL-18). Importantly, the IL-18 armoring strategy has now reached the clinic: Svoboda and colleagues reported a Phase 1 trial of an IL-18-secreting anti-CD19 CAR-T product (huCART19-IL18) in patients with relapsed or refractory lymphoma after prior CAR-T failure, demonstrating durable responses at low cell doses without unexpected toxicity [35]. Although this trial was conducted in a hematologic malignancy rather than a solid tumor, it provides the first clinical proof of concept that cytokine armoring with IL-18 is feasible and biologically active in patients, lending clinical weight to the preclinical solid tumor rationale described above.

Nguyen and colleagues demonstrated that cooperative armoring with membrane-tethered IL-15 and IL-21 achieves greater efficacy than either cytokine alone, as the two signals converge on complementary aspects of T-cell fitness: IL-15 enhances proliferative capacity while IL-21 modulates effector differentiation [36].

### Dominant-negative receptors: Microenvironmental shields

TGF-β is the master regulator of immunosuppression in many solid TMEs. The dominant-negative TGF-β receptor type II (dnTGF-βRII) retains the extracellular binding domain but lacks the intracellular kinase domain, functioning as both a decoy sequestering soluble TGF-β and a competitive inhibitor on the T-cell surface. The clinical validation of this approach in C-CAR031 is detailed in a subsequent section.

Analogous strategies have been applied to the PD-1 axis. Cherkassky and colleagues at Memorial Sloan Kettering demonstrated that expression of a PD-1 dominant-negative receptor on mesothelin-targeting CAR-T cells restores anti-tumor function in mesothelioma models [37]. Working at the University of Pennsylvania, Liu X and colleagues developed a PD-1/CD28 chimeric switch receptor that converts the inhibitory PD-1 signal into a costimulatory CD28 signal, demonstrating augmented efficacy of second-generation CAR-T cells in mouse models bearing human solid tumors [38]. In a separate study, Liu H and colleagues in China, with collaborators at Duke University, evaluated a CD19-directed CAR-T product incorporating a PD-1/CD28 switch receptor in a Phase 1 clinical trial, reporting an ORR of 58.8% and a CR rate of 41.2% in patients with PD-L1-positive B-cell lymphoma [39]. These are independent studies from different groups employing different models: the switch-receptor architecture was established preclinically in solid tumor xenografts [38], whereas the available clinical experience derives from a hematologic malignancy [39]. Together they provide proof-of-concept for the switch receptor architecture in solid tumors where PD-L1 expression is prevalent.

### Chemokine receptor engineering for tumor trafficking

A CAR-T cell that cannot reach the tumor is functionally irrelevant regardless of its intrinsic potency. The engineering of chemokine receptors addresses this trafficking bottleneck. Craddock and colleagues first demonstrated that co-expression of CCR2b on GD2-targeting CAR-T cells increased tumor homing more than 10-fold [40]. Moon and colleagues at the University of Pennsylvania extended this approach to mesothelin-targeting CARs, achieving a 12.5-fold increase in T-cell infiltration of mesothelioma tumors via CCR2 expression [41]. Di Stasi and colleagues earlier demonstrated that CCR4 co-expression enhanced homing in a Hodgkin lymphoma model [42]. These findings establish chemokine receptor engineering as a general strategy for overcoming the trafficking barrier, which is particularly relevant for tumors that secrete specific chemokine gradients but lack the complementary receptors on standard T cells.

### Hypoxia-responsive and ROS-resistant engineering

**Figure 2:**
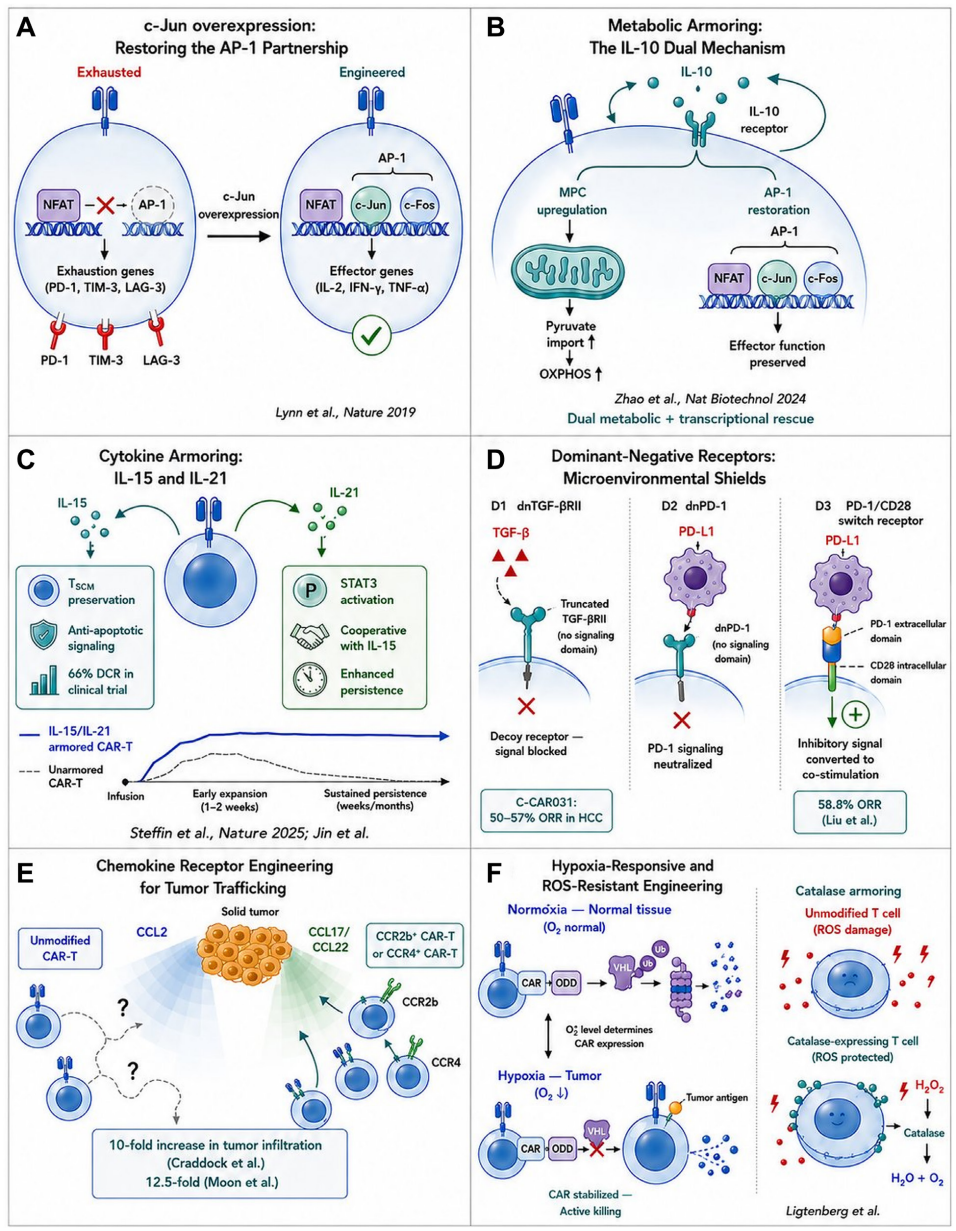
Engineering strategies for resilience-based CAR-T design. Overview of the major engineering approaches transitioning from the “Potency Era” to the “Resilience Era.” (**A**) c-Jun overexpression restores AP-1 partnership with NFAT, preventing exhaustion program initiation. (**B**) Metabolic armoring via autocrine IL-10 promotes mitochondrial fitness through MPC-dependent pyruvate import. (**C**) Cytokine armoring with IL-15 and IL-21 preserves stem cell memory phenotype. (**D**) Dominant-negative receptors (dnTGF-βRII, dnPD-1, PD-1/CD28 switch) neutralize suppressive signals. (**E**) Chemokine receptor co-expression (CCR2b, CCR4) enhances tumor trafficking. (**F**) Hypoxia-responsive CARs (HIF-ODD, HypoxiCAR) restrict activity to the tumor microenvironment.

The hypoxic tumor core presents both a challenge and an engineering opportunity. Juillerat and colleagues developed the first oxygen-sensitive CAR by fusing the CAR construct to the HIF-1α oxygen-dependent degradation domain (ODD), creating a CAR that is expressed only under hypoxic conditions [43]. Kosti and colleagues refined this into the HypoxiCAR, combining a hypoxia-response element (HRE) promoter with ODD fusion for dual-level oxygen sensing [44]. Such constructs offer an inherent safety mechanism: the CAR is active only in the hypoxic tumor core and inactive in normoxic normal tissues. Separately, Ligtenberg and colleagues demonstrated that co-expression of catalase protects CAR-T cells from reactive oxygen species (ROS)-mediated dysfunction, preserving cytotoxicity in oxidative stress environments characteristic of myeloid-rich TMEs [45]. These engineering strategies are summarized in Figure 2.

## SYNTHETIC BIOLOGY AND ADVANCED TARGETING PLATFORMS

### SynNotch and logic-gated circuits

The seminal breakthrough in synthetic T-cell logic was the development of synthetic Notch (synNotch) receptors by Roybal and Lim. synNotch receptors comprise an extracellular recognition domain, the Notch core regulatory region, and an intracellular transcriptional activator. Upon ligand engagement, the Notch core is cleaved, releasing the transcriptional activator to drive expression of a user-defined gene cassette (such as a CAR, cytokine, or therapeutic payload) [46, 47]. Critically, this architecture enables AND-gate logic: a priming antigen (detected by synNotch) must be present to induce expression of the CAR, which then activates only upon recognition of a second antigen. Williams and colleagues subsequently demonstrated that transcriptional linking of multiple receptors enables precise T-cell recognition programs with enhanced specificity [48]. Hyrenius-Wittsten and colleagues showed that synNotch circuits enhance solid tumor recognition and promote persistent anti-tumor activity in mouse models [49].

Drug-gated CAR platforms represent a complementary approach. Labanieh and colleagues developed SNIP CARs (Signal Neutralization by an Inhibitable Protease), where CAR surface expression requires the presence of a small-molecule protease inhibitor [50]. This provides an external pharmacological control over CAR activity, providing a reversible on/off switch controllable by drug administration and withdrawal.

The Tmod platform applies NOT-gate Boolean logic. An activating CAR recognizes a tumor-associated antigen while a blocking receptor recognizes HLA-A*02 present on normal cells but lost on tumor cells through loss of heterozygosity [51]. The EVEREST-2 trial (A2B694) has reported the first-ever complete response to logic-gated CAR-T in a patient with NSCLC [52].

### Base editing and epigenetic engineering

CRISPR base editing, which introduces precise single-nucleotide changes without double-strand breaks, has become a groundbreaking tool for CAR-T engineering. Chiesa and colleagues at Great Ormond Street Hospital reported the first-in-human data on base-edited CAR-T (BE-CAR7) for T-cell acute lymphoblastic leukemia [53], with expanded phase 1 results subsequently published [54]. While these applications are in hematologic malignancies, the technology has direct relevance to solid tumor engineering: base editing enables multiplex gene disruption (e.g., simultaneous knockout of TCR, HLA, and checkpoint genes) with reduced risk of chromosomal translocations compared to nuclease-based CRISPR.

Epigenetic editing offers a distinct therapeutic paradigm. Beyond the DNMT3A knockout data discussed earlier, Fraietta and colleagues reported a landmark observation: a patient with chronic lymphocytic leukemia achieved a sustained complete remission when the lentiviral CAR vector fortuitously integrated into and disrupted the TET2 gene, resulting in massive clonal expansion of a single CAR-T cell with enhanced effector function [55]. This serendipitous finding has motivated deliberate TET2 disruption as an engineering strategy, though the safety of intentional epigenetic modifier disruption remains under investigation.

### Allogeneic and off-the-shelf platforms

**Figure 3:**
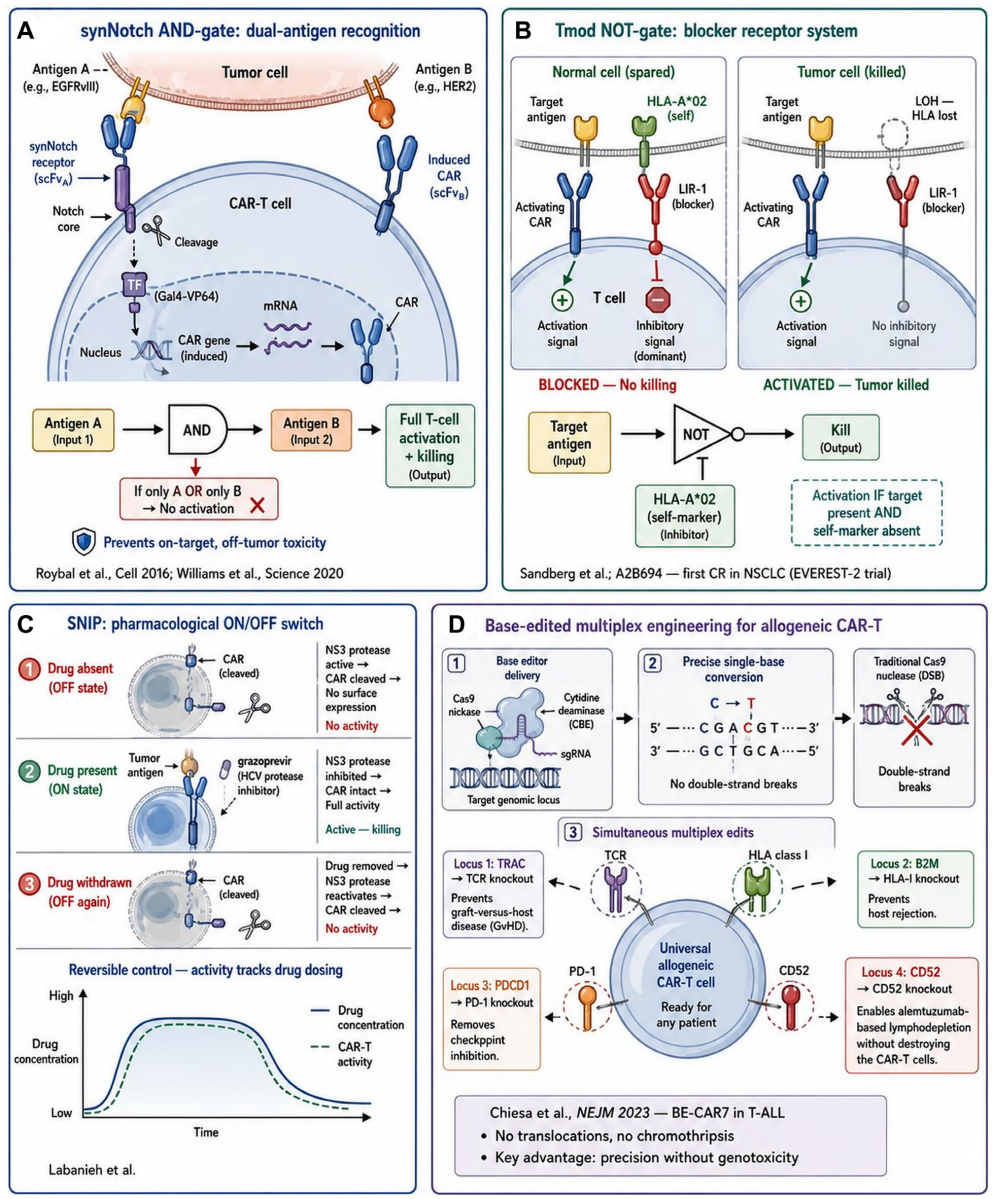
Synthetic biology approaches to CAR-T engineering. Schematic of advanced synthetic biology architectures. (**A**) synNotch AND-gate: a synNotch receptor recognizing Antigen A drives transcription of a CAR targeting Antigen B, requiring both antigens for full activation. (**B**) Tmod NOT-gate: an activating CAR is overridden by a blocker receptor recognizing a “self” antigen (e.g., HLA-A\*02) lost on tumor cells. (**C**) SNIP CARs: drug-gated expression requiring a small-molecule protease inhibitor for CAR surface display. (**D**) Base-edited multiplex engineering: simultaneous disruption of TCR, HLA, and checkpoint genes via cytidine or adenine base editors.

Autologous CAR-T manufacturing imposes inherent limitations: variable starting material quality, manufacturing delays, and scalability constraints. Allogeneic platforms seek to address these through “off-the-shelf” products derived from healthy donors. Benjamin and colleagues reported the first allogeneic CAR-T data with UCART19 (Cellectis/Servier), using TALEN-mediated TCR disruption to prevent graft-versus-host disease [56]. CRISPR-based approaches have followed, including Locke and colleagues’ report on ALLO-501/501A (Allogene Therapeutics) [57]. For solid tumors, allogeneic platforms are especially appealing since the manufacturing timeline (typically 3–6 weeks for autologous products) frequently cannot keep pace with disease advancement. These synthetic biology approaches are illustrated in Figure 3.

## THE 2024–2025 CLINICAL INFLECTION POINT

The engineering advances described above have now translated into tangible clinical outcomes. We examine the three landmark programs and contextualize them with historical lessons from earlier solid tumor trials.

### Central nervous system: GD2 and B7-H3

The CNS has traditionally functioned as a protected site for tumors. For H3K27M-mutated diffuse midline gliomas (DMGs), median survival remains below one year with radiation alone. The H3K27M mutation drives uniform, high-density GD2 expression, approaching the antigen uniformity of CD19 in B-ALL. The trial (NCT04196413) employed intravenous dosing followed by intracerebroventricular (ICV) infusions via Ommaya reservoir [8, 9]. Among evaluable patients, four achieved major tumor volume reductions of 52%, 54%, 91%, and 100%, with one CR sustained beyond 30 months. The trial also defined Tumor Inflammation-Associated Neurotoxicity (TIAN) as a novel toxicity syndrome [58].

Building on this paradigm, BrainChild Bio’s BCB-276, targeting B7-H3 (CD276), received FDA Breakthrough Therapy Designation in April 2025 for DIPG [59]. The BrainChild-03 trial (NCT04185038) delivered 253 ICV doses across 21 patients, with median OS of 19.8 months from diagnosis and three patients alive at 44–52 months [60]. BCB-276 represents the first CAR-T therapy to receive BTD for a solid tumor indication.

### Gastric cancer: The first randomized superiority

CLDN18.2 is a tight junction protein exposed on tumor cells due to loss of epithelial polarity. Phase 1 data (CT041-CG4006) demonstrated an interim ORR of 57.1% in the gastric cohort [61], with final results reporting 38.8% ORR and median OS of 8.8 months [62]. The pivotal Phase 2 randomized trial (CT041-ST-01) randomized 156 patients (2:1) to satri-cel versus physician’s choice [10, 11]. PFS was 3.25 versus 1.77 months (HR 0.37, *p* < 0.0001); OS was 7.92 versus 5.49 months (HR 0.69, *p* = 0.04); confirmed ORR was 22% versus 4%. CRS occurred in 95.5% but was predominantly grade 1–2. CARsgen received FDA RMAT designation in January 2022 [63] and Chinese NMPA Breakthrough Therapy Designation in March 2025.

### Hepatocellular carcinoma: The triumph of armoring

Shi and colleagues reported on unarmored GPC3-CAR-T in HCC, observing an ORR of approximately 15% and 3-year OS rate of 10.5% [13]. C-CAR031 (AbelZeta Pharma/AstraZeneca) incorporated dnTGF-βRII armor. Two data cuts demonstrated ORR of 50.0% (January 2024, *n* = 22) and 56.5% (March 2024, *n* = 23), with 57.1–75.0% at recommended dose level 4, DCR of 90.9–91.3%, and zero DLTs or ICANS [12]. We note that these GPC3-CAR-T efficacy data derive from non-peer-reviewed conference abstracts; full peer-reviewed publication of these outcomes is awaited and will be necessary for definitive interpretation. This program nevertheless constitutes a natural “A/B experiment”: same target, same disease, sole variable being the dnTGF-βRII modification. AstraZeneca’s acquisition of AbelZeta’s remaining China rights for up to $630 million in January 2026 signals industry confidence.

### Historical lessons: HER2, EGFRvIII, and mesothelin

The current successes must be contextualized against sobering historical precedents. Morgan and colleagues at the NCI reported a fatal case in 2010 when a HER2-targeting CAR-T cell (using a high-affinity trastuzumab-derived scFv) recognized low-level HER2 expression on lung epithelium, triggering fulminant cytokine release and respiratory failure [64]. This case established the on-target/off-tumor toxicity paradigm and fundamentally redirected the field toward reduced-affinity scFvs and logic-gated safety mechanisms. Subsequent HER2-CAR-T programs using optimized constructs and lower-affinity binders have demonstrated acceptable safety profiles: Ahmed and colleagues reported a Phase 1 trial in sarcoma [65] and GBM [66], with the HEROS 2.0 trial further establishing feasibility with lymphodepletion [67].

EGFRvIII-targeting in glioblastoma provided the definitive demonstration of antigen escape. O’Rourke and colleagues showed that 5 of 7 evaluable patients had reduced EGFRvIII expression after CAR-T treatment [68], illustrating how single-antigen targeting exerts evolutionary selection pressure on heterogeneous tumors. This finding directly motivated the multi-antigen and logic-gated approaches described earlier.

Mesothelin-targeting CARs have explored delivery innovation. Adusumilli and colleagues at Memorial Sloan Kettering demonstrated that intrapleural delivery of mesothelin-CAR-T combined with pembrolizumab achieves locoregional disease control in malignant pleural mesothelioma [69]. Beatty and Haas at the University of Pennsylvania explored both mRNA-based [70] and lentiviral [71] mesothelin CAR-T constructs, establishing safety and detecting evidence of anti-tumor activity. Building on these foundations, Barber-Rotenberg and colleagues recently reported a Phase 1 study of autologous T cells bearing fully humanized chimeric antigen receptors targeting mesothelin in patients with lung adenocarcinoma, ovarian cancer, and mesothelioma [72]. This trial established the safety and feasibility of the fully human binder, but the best overall response was stable disease in 12 of 20 patients, with a maximum target lesion reduction of 41% and no objective responses. The result therefore reflects a largely negative efficacy outcome and underscores that scFv humanization alone, in the absence of armoring or microenvironmental engineering, has not been sufficient to drive clinical responses against these solid tumors. This iterative refinement of mesothelin-targeting constructs over more than a decade exemplifies how individual safety and efficacy lessons translate into successive engineering generations.

### Novel targets: The CARVac paradigm (CLDN6)

**Figure 4:**
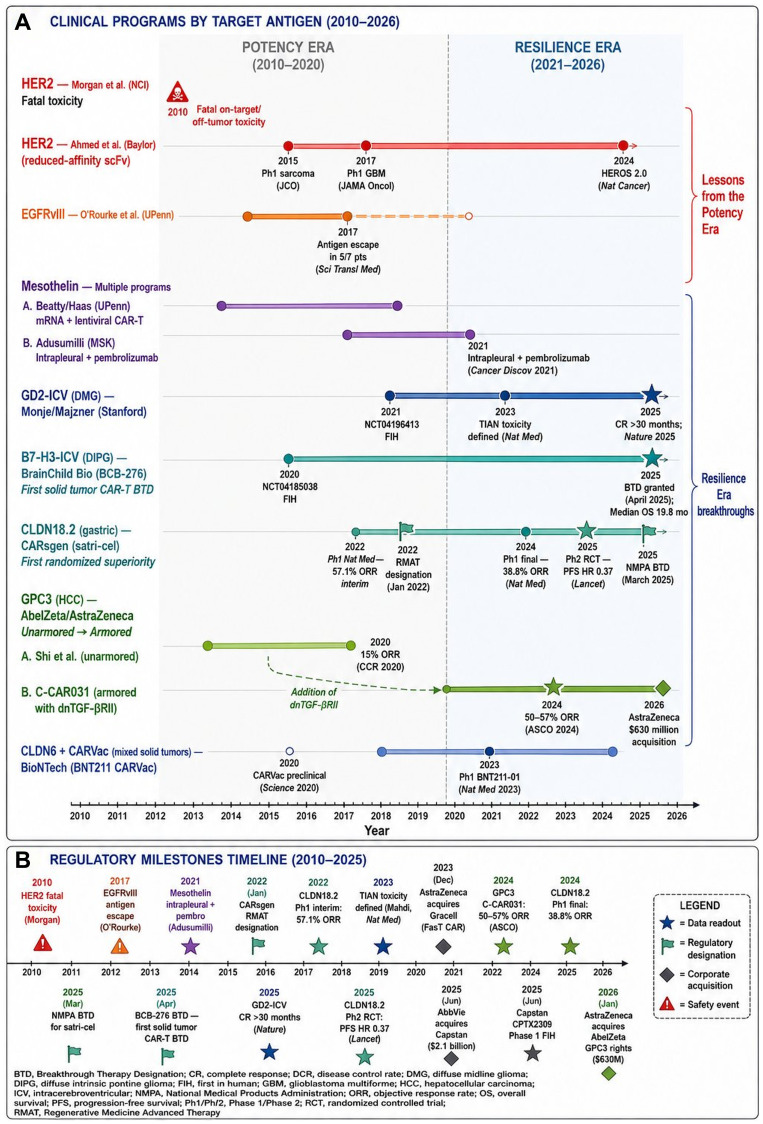
Clinical trial landscape: Solid tumor CAR-T programs 2015–2025. Timeline of key clinical trials and regulatory milestones in solid tumor CAR-T therapy. (**A**) Top panel: select programs colored by target antigen (GD2, B7-H3, CLDN18.2, GPC3, Mesothelin, HER2, EGFRvIII, CLDN6). (**B**) Bottom panel: regulatory milestones including BTD, RMAT designations, and pivotal trial readouts. Shaded regions indicate the “Potency Era” (2010–2020) and “Resilience Era” (2021–present).

BioNTech’s BNT211 program introduces a conceptually novel approach: combining CLDN6-targeting CAR-T cells with an mRNA vaccine encoding the same CLDN6 antigen. Reinhard and colleagues demonstrated preclinically that this “CARVac” approach periodically re-stimulates and expands CAR-T cells *in vivo*, mimicking repeated antigen boosting [73]. Mackensen and colleagues reported the Phase 1 BNT211-01 clinical data, establishing safety and manageable toxicity in relapsed or refractory solid tumors [74]. It should be emphasized, however, that this early-phase trial did not provide clear evidence that the RNA vaccine component itself conferred a discrete clinical benefit; the observed activity cannot be confidently attributed to the vaccine-driven amplification mechanism as distinct from the CAR-T cells alone. The CARVac concept therefore remains a mechanistically attractive but clinically unproven approach to the persistence challenge: in principle it provides periodic exogenous stimulation to maintain effector function, but this proposed advantage awaits confirmation in controlled studies. The clinical trial landscape is summarized in Figure 4.

## THE TUMOR MICROENVIRONMENT: CELLULAR BARRIERS AND TARGETED COUNTERMEASURES

### Myeloid-derived suppressor cells

MDSCs represent a heterogeneous population of immature myeloid cells that directly suppress CAR-T function. Tumino and colleagues provided the first clinical demonstration of MDSC-mediated CAR-T resistance, showing that polymorphonuclear MDSCs impair GD2-CAR-T efficacy in neuroblastoma patients [75]. Mechanistic approaches to overcome this barrier include CSF1R blockade, recently demonstrated by Stahl and colleagues to synergize with CD19-CAR-T in aggressive B-cell lymphoma [76], building on foundational work by Zhu and colleagues showing that CSF1/CSF1R blockade reprograms tumor-infiltrating macrophages [77]. An alternative engineering approach was reported by Nalawade and colleagues, who developed CAR-T cells co-expressing a TR2-targeting moiety that selectively depletes MDSCs via TRAIL receptor 2 [78].

### Cancer-associated fibroblasts

Cancer-associated fibroblasts (CAFs) produce the dense desmoplastic stroma that physically excludes T cells. Lo and colleagues demonstrated that FAP-targeting CAR-T cells disrupt tumor-promoting desmoplasia [79], and Xiao and colleagues subsequently showed that FAP-CAR pretreatment enables subsequent mesothelin-CAR-T and anti-PD-1 efficacy by removing the physical barrier to T-cell infiltration [80]. This sequential approach (first deplete the stroma, then attack the tumor) offers a promising therapeutic approach for desmoplastic malignancies such as pancreatic ductal adenocarcinoma.

### The adenosine pathway

The CD73/CD39/A2A receptor axis constitutes a major immunosuppressive pathway. CD39 and CD73 expressed on tumor cells and Tregs convert extracellular ATP to adenosine, which signals through the A2A receptor on T cells. Downstream of A2AR engagement, intracellular cAMP rises and activates Protein Kinase A (PKA), which localizes to the immune synapse and inhibits effector function. Three distinct engineering strategies have been developed to interrupt this pathway. Giuffrida and colleagues demonstrated that CRISPR-mediated deletion of the A2A receptor enhances CAR-T efficacy [81], establishing the receptor-level genetic approach. Masoumi and colleagues subsequently showed that combined genetic and pharmacological targeting of A2AR improves the function of anti-mesothelin CAR T cells [82], expanding the receptor-level strategy. Newick and colleagues developed a complementary approach targeting the downstream signaling node: by expressing the regulatory subunit I anchoring disruptor (RIAD) peptide within CAR-T cells, they displaced PKA from the immune synapse, augmenting CAR-T trafficking and antitumor efficacy [83]. Together, these three strategies illustrate that the adenosine-A2AR-cAMP-PKA pathway can be interrupted at multiple nodes (receptor deletion, receptor-level genetic-pharmacological combination, and downstream kinase displacement), offering complementary engineering options for solid tumor CAR-T design.

### Combination strategies: Oncolytic viruses and innate immune agonists

**Figure 5:**
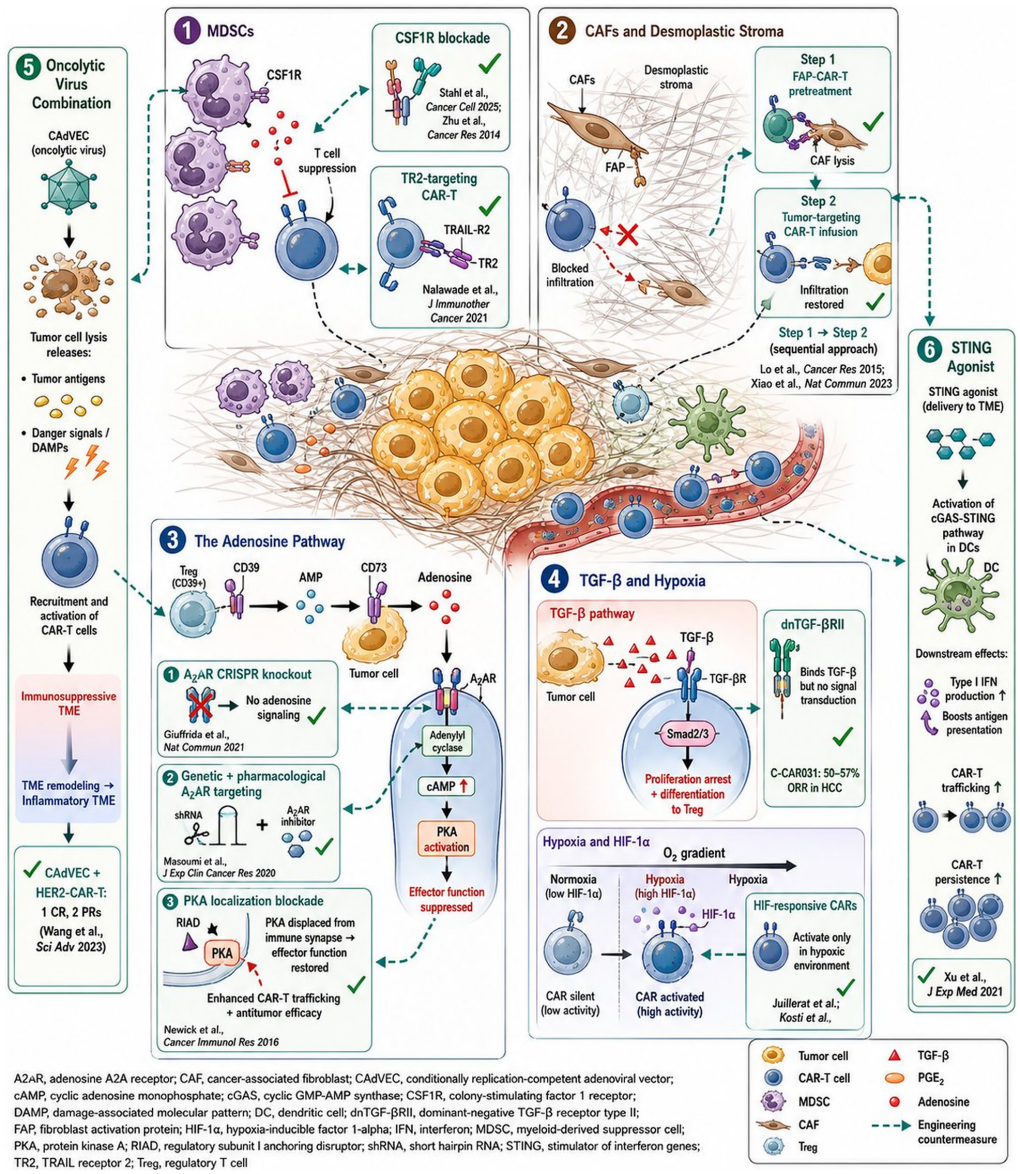
Tumor microenvironment cellular interactions and engineering countermeasures. Schematic of the cellular components of the immunosuppressive TME and their engineering countermeasures. MDSCs are targeted via CSF1R blockade or TRAIL receptor 2-targeting. CAFs and desmoplastic stroma are addressed through FAP-CAR-T pretreatment. The adenosine pathway (CD73/CD39/A2AR) is countered by A2AR knockout, combined genetic and pharmacological A2AR targeting, and downstream PKA localization blockade via the RIAD peptide. Hypoxia is exploited via HIF-responsive CARs. TGF-β is neutralized by dnTGF-βRII. Combination approaches include oncolytic viruses (CAdVEC) for TME remodeling and STING agonists for innate immune activation.

Combination of CAR-T with oncolytic viruses offers a conceptually elegant strategy: the virus lyses tumor cells (releasing antigen and danger signals), while remodeling the TME from immunosuppressive to inflammatory. The CAdVEC platform (binary oncolytic adenovirus) demonstrated preclinical synergy with HER2-CAR-T in pancreatic cancer models [84]. Wang and colleagues reported early clinical data showing 1 CR and 2 PRs among 4 treated patients in a combined CAdVEC/HER2-CAR-T approach [85]. STING agonists represent another avenue: Xu and colleagues demonstrated that STING activation promotes CAR-T trafficking and persistence in breast cancer models [86]. The TME cellular components and their engineering countermeasures are depicted in Figure 5.

### Systemic dimensions of the tumor–host interaction

Beyond the local cellular and molecular barriers of the TME, contemporary understanding increasingly recognizes that solid tumors influence whole-body homeostasis through neuroendocrine-immune pathways. Slominski and colleagues have synthesized evidence that cancers hijack systemic regulation via the hypothalamic-pituitary-adrenal (HPA) axis and sympathetic nervous system, generating sustained glucocorticoid and catecholamine elevations that broadly modulate immune tone, inflammation, and metabolism [87]. This systemic dysregulation has direct, mechanistically supported implications for CAR-T persistence and fitness.

Bucsek and colleagues demonstrated that β-adrenergic signaling in mice housed at standard temperatures suppresses an effector phenotype in CD8+ T cells and undermines checkpoint inhibitor therapy, with thermoneutral housing or β-blocker administration restoring T-cell function [88]. Qiao and colleagues subsequently showed that β-adrenergic signaling specifically blocks CD8+ T-cell metabolic reprogramming during activation, identifying a mechanism by which sympathetic tone undermines the oxidative-glycolytic transitions required for cytotoxic function [89]. Cole and Sood synthesized the broader translational evidence linking β-adrenergic signaling to cancer progression and therapeutic outcomes [90], while Kokolus and colleagues reported that β-blocker use correlates with improved overall survival in metastatic melanoma patients and enhances immunotherapy efficacy in preclinical models [91]. Most directly relevant to combination strategies, Gandhi and colleagues reported a Phase I clinical trial of propranolol combined with pembrolizumab in advanced melanoma, demonstrating safety, tolerability, and preliminary evidence of antitumor activity [92].

For CAR-T cell therapy, these findings suggest that the systemic neuroendocrine state of patients with advanced solid malignancies, often characterized by chronic stress, cachexia, and HPA axis activation, may impose additional pressure on infused T cells beyond the local TME. Pre-conditioning regimens that account for systemic neuroendocrine dysregulation, β-blocker co-administration, and attention to circadian and stress-related variables represent emerging considerations that have received insufficient attention in engineering-focused CAR-T literature. Acknowledging this systemic dimension reframes the therapeutic challenge: optimizing CAR-T outcomes requires not only engineering the cell and modifying the local niche, but also addressing the broader physiological context into which the cell is delivered.

## THE MICROBIOME: AN UNANTICIPATED DETERMINANT OF OUTCOMES

Smith and colleagues demonstrated that exposure to broad-spectrum antibiotics, specifically piperacillin-tazobactam, imipenem-cilastatin, and meropenem (the P-I-M group), in the weeks preceding CAR-T infusion significantly degraded survival and increased ICANS severity (OS HR 2.58; 95% CI 1.68–3.98) [93]. Higher abundances of *Ruminococcus*, *Bacteroides*, and *Faecalibacterium* were associated with day-100 complete response. Stein-Thoeringer and colleagues extended these findings, identifying cefepime as an additional high-risk agent [94].

The mechanistic link centers on short-chain fatty acids (SCFAs), principally butyrate. Luu and colleagues demonstrated that pentanoate and butyrate augment CAR-T anti-tumor activity through mTOR signaling and class I HDAC inhibition [95], which is particularly intriguing given the role of HDAC-mediated regulation in the exhaustion program. The clinical significance is evident: antibiotic stewardship should be acknowledged as a cornerstone of CAR-T management as critical as lymphodepletion conditioning.

## MANUFACTURING AND ACCESS: FROM WEEKS TO *IN VIVO* GENERATION

Locke and colleagues demonstrated that vein-to-vein (V2V) time exceeding 40 days independently predicts worse outcomes after axi-cel (24-month OS 38% vs. 53%, HR 1.33) [96]. The T-cell memory subset composition at infusion is equally critical: Gattinoni and colleagues discovered human T stem cell memory (T_SCM) cells as a population with superior proliferative and persistence capacity [97, 98], and Meyran and colleagues demonstrated that T_SCM-like CAR-T cells exhibit improved persistence and tumor control [99].

Next-day manufacturing platforms address both the temporal and biological limitations. The FasT CAR platform reduces manufacturing to 22–36 hours [100], preserving a “young,” stem-like T-cell state.

### *In Vivo* CAR-T generation: From concept to clinical trial

The logical endpoint of manufacturing innovation is *in vivo* CAR-T generation, in which CAR-encoding genetic material is delivered to T cells within the patient, eliminating *ex vivo* manufacturing entirely. Two main delivery modalities have advanced: lipid nanoparticle (LNP)-mediated mRNA delivery and lentiviral vector-mediated stable integration.

The foundational preclinical demonstration of *in vivo* CAR-T generation came from Pfeiffer and colleagues, who used CD8-targeted lentiviral vectors to generate human CD19-CAR T cells directly *in vivo*, producing B-cell depletion and signs of cytokine release syndrome in humanized mice [101]. Agarwal and colleagues extended this approach to selective generation of CAR T cells in human CD4+ lymphocytes via receptor-targeted lentiviral particles [102]. Hamilton and colleagues subsequently developed enveloped delivery vehicles for *in vivo* human T-cell engineering, demonstrating the feasibility of CRISPR-based genome editing *in vivo* [103]. Conceptual proof-of-concept for transient mRNA-based *in vivo* CAR-T generation was provided by Rurik and colleagues, who used LNP-delivered modified mRNA to generate fibroblast-activation-protein-targeting CAR T cells *in vivo* for the treatment of cardiac fibrosis [104].

These preclinical advances have catalyzed first-in-human clinical trials. Capstan Therapeutics initiated the first-in-human Phase 1 trial of their tLNP-based platform (CPTX2309) in June 2025 [105], with subsequent acquisition by AbbVie for $2.1 billion [106]. In parallel, three lentiviral-based *in vivo* CAR-T programs have entered clinical evaluation. Umoja Biopharma’s UB-VV111, an in situ CAR-T platform using the VivoVec lentiviral delivery system to generate CD19-directed CAR T cells, received FDA IND clearance and entered first-in-human evaluation in the INVICTA-1 trial (NCT06528301), with FDA Fast Track Designation granted in September 2025 [107]. Interius BioTherapeutics dosed the first patient with INT2104, an *in vivo* CD20-directed lentiviral platform, in October 2024 under the INVISE trial in Australia (NCT06539338) [108]. EsoBiotec dosed the first patient with ESO-T01, an *in vivo* BCMA-directed CAR-T candidate using the ENaBL lentiviral platform, in January 2025 for relapsed/refractory multiple myeloma (NCT06691685) [109]. These programs collectively represent the first clinical translation of *in vivo* lentiviral CAR-T generation and signal a potential shift away from *ex vivo* cell manufacturing entirely. While early clinical safety and efficacy data remain limited and non-peer-reviewed at the time of writing, the parallel emergence of multiple programs across LNP-mRNA and lentiviral modalities suggests that *in vivo* CAR-T generation may transition from concept to clinical reality within the current decade.

**Figure 6:**
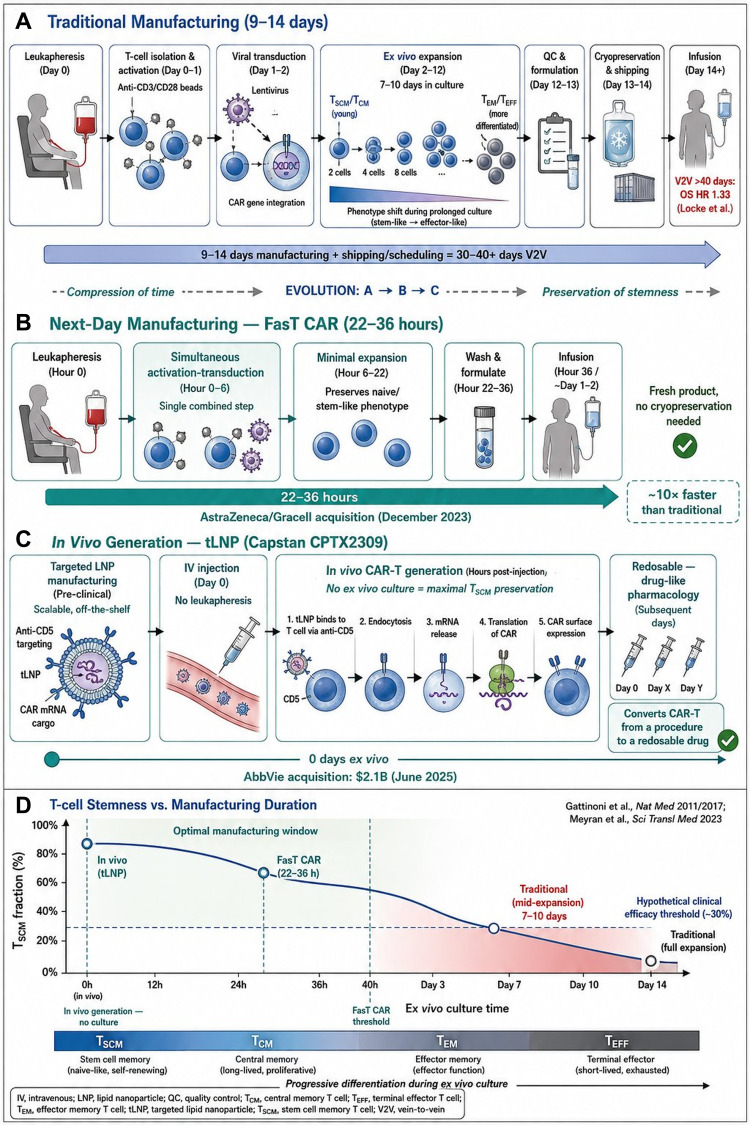
Evolution of CAR-T manufacturing: from weeks to *in vivo* generation. Comparative timeline of CAR-T manufacturing paradigms. (**A**) Traditional manufacturing (9–14 days) involves leukapheresis, activation, transduction, expansion, and reinfusion. (**B**) Next-day manufacturing (FasT CAR, 22–36 hours) employs concurrent activation-transduction. (**C**) *In vivo* generation (Capstan CPTX2309, Umoja UB-VV111, Interius INT2104, EsoBiotec ESO-T01) eliminates *ex vivo* manufacturing entirely using targeted lipid nanoparticles or receptor-targeted lentiviral particles. (**D**) Bottom panel: biological consequences of manufacturing duration, illustrating the inverse relationship between *ex vivo* culture time and T cell memory/stemness phenotype (TSCM fraction).

The evolution of manufacturing platforms is illustrated in Figure 6.

## AN INTEGRATED FRAMEWORK FOR SOLID TUMOR CAR-T DEVELOPMENT

The synthesis of evidence presented in this review enables the formulation of a tiered, evidence-based framework for rational next-generation development (Table 1).

**Table 1:** Proposed tiered framework for solid tumor CAR-T development

Tier	Principle	Key strategy	Validation	Status
**1**	Delivery	Locoregional (ICV, IP) for sanctuary sites	GD2-ICV [8, 9]; B7-H3 [60]	Ph1 CR >30 mo
**2**	Resilience	Armoring (dnTGF-β, IL-10, IL-15, IL-18)	C-CAR031 [12]; Steffin [30]; Zhao [29]; Chmielewski [32]	Ph1 50–57% ORR
**3**	Logic	synNotch AND, Tmod NOT, SNIP drug-gated	A2B694 [52]; BNT211 CARVac [73, 74]	Ph1 first CR
**4**	System	Microbiome, conditioning, TSCM, neuroendocrine modulation	Smith [93]; Luu [95]; Slominski [87]; Bucsek [88]	Correlational; prospective needed

Table 1 summarizes the proposed tiered framework for solid tumor CAR-T development across four hierarchical engineering principles (Delivery, Resilience, Logic, and System), with key strategies, validating studies, and clinical status for each tier. Tier 1: Delivery is Prerequisite. For sanctuary sites (CNS, pleura, peritoneum), locoregional delivery is mandatory. The GD2-ICV success versus the limited systemic experience for the same target provides direct evidence. Tier 2: Resilience is Required. For immunosuppressive TMEs (HCC, pancreatic cancer), armoring is non-negotiable. dnTGF-βRII and IL-10/IL-15 armoring have demonstrated superiority over unarmored constructs. Tier 3: Targeting is Logic. For tumors where the antigen is shared with vital tissues, logic-gated architectures are required. Tier 4: The System is the Therapy. Microbiome composition, manufacturing duration, T-cell phenotype at infusion, conditioning regimen, and the broader systemic neuroendocrine context are integral to the therapeutic product.

### Operationalizing the integrated design concept

Translating this framework into rational construct selection requires biomarker-driven decision criteria that pair tumor biology with engineering strategy. Tumor antigen heterogeneity assessed via immunohistochemistry or spatial profiling should motivate the choice between single-target and logic-gated (AND/OR-gate) architectures. Quantification of stromal density and TGF-β pathway activation (for example, by Smad2/3 phosphorylation or TGF-β signature scores) should inform the selection of dominant-negative or trafficking-enhanced constructs over conventional CARs. Hypoxia signatures or HIF-1α staining patterns may identify tumors that benefit from hypoxia-responsive CAR designs. T-cell intrinsic predictors, including baseline T_SCM fraction, mitochondrial fitness, and exhaustion gene signatures in the apheresis product, should guide the use of c-Jun, IL-10, or DNMT3A-disrupted constructs versus standard manufacturing. This biomarker-to-engineering matching represents an aspirational standard; prospective trials with embedded correlative biology are needed to validate which decision rules translate into clinical benefit.

### Limitations and unknowns

Despite the conceptual coherence of this framework, important limitations and unknowns warrant explicit acknowledgement.

#### Durability of multiplex edits

While base editing reduces the translocation risk associated with nuclease-based CRISPR multiplexing [110–112], the long-term genomic stability of multiplex-edited CAR-T cells in humans is not yet established. Sasu and colleagues reported a chromosomal alteration detected after infusion of gene-edited allogeneic CAR T cells, underscoring the importance of long-term safety surveillance [113]. The risk profile of editing three to four loci simultaneously, as exemplified by the Diorio quadruple-edited construct [110], remains under investigation in expanded clinical cohorts. Authoritative recent reviews of these safety considerations have been provided by Qasim [114].

#### Safety of sustained cytokine armoring

IL-15, IL-18, and IL-10 armored constructs deliver continuous cytokine exposure to the infused T cells and, depending on construct design, to bystander cells. The risk of autonomous proliferation, ectopic immune activation, and cytokine-release-related toxicity must be balanced against the substantial efficacy gains observed [29, 30, 32–34]. Inducible safety switches such as the iCasp9 system [115] provide a mitigation strategy, but the optimal trigger thresholds and clinical management algorithms remain to be defined for armored constructs in solid tumor applications.

#### Manufacturability constraints

Advanced CAR-T constructs increasingly require multiplex gene insertion and disruption, raising the bar for transduction efficiency, cell yield, and manufacturing reproducibility. The translation of complex engineered constructs from academic laboratories to commercial-scale Good Manufacturing Practice (GMP) processes is non-trivial, and regulatory pathways for *in vivo* CAR-T generation platforms, where the patient’s body becomes the manufacturing vessel, are still being defined.

These limitations do not negate the framework but rather identify the empirical and translational work required to convert it into validated clinical practice.

## CONCLUSIONS AND FUTURE PERSPECTIVES

The solid tumor barrier no longer represents a monolithic obstacle. Rather, it represents a collection of discrete, definable engineering challenges (trafficking, metabolic fitness, epigenetic stability, target specificity, and systemic context) that are being methodically tackled [7, 26]. The 57% ORR in gastric cancer, the 50–57% ORR in hepatocellular carcinoma, and the durable complete responses in diffuse midline glioma are the first clinical returns of a rational, resilience-based engineering era.

Several emerging avenues warrant specific focus. First, the convergence of metabolic and epigenetic armoring: IL-10’s dual mechanism (MPC-dependent metabolic support and AP-1 restoration) indicates that future designs should incorporate multiple protective mechanisms instead of depending on isolated modifications. Second, the CARVac paradigm (periodic *in vivo* re-stimulation via mRNA vaccination) offers a mechanistically appealing route to addressing the persistence limitation without permanent genetic modification, though its independent clinical contribution remains to be demonstrated. Third, *in vivo* CAR-T generation via targeted LNPs and lentiviral platforms has the potential to transform CAR-T from a procedure into a repeatedly administrable therapeutic, broadening access to what has been a therapy restricted to specialized facilities. Fourth, broader recognition of systemic neuroendocrine-immune regulation as a determinant of CAR-T fitness may motivate adjunctive pharmacological strategies (β-blockade, stress modulation) as part of comprehensive CAR-T management.

The path forward requires not merely applying hematologic paradigms to solid tumors, but fundamentally reconceptualizing the T cell as a drug product that must be delivered to the right compartment, armored against suppression, and integrated into a holistic therapeutic system that includes manufacturing optimization, microbiome stewardship, systemic physiological context, and rational combination strategies.

## Abbreviations

ACT: adoptive cell transfer
ATAC-seq: Assay for Transposase-Accessible Chromatin with sequencing
B-ALL: B-cell acute lymphoblastic leukemia
BBB: blood–brain barrier
BCMA: B-cell maturation antigen
BTD: Breakthrough Therapy Designation
CAF: cancer-associated fibroblast
cAMP: cyclic adenosine monophosphate
CAR-T: chimeric antigen receptor T cell
CNS: central nervous system
CR: complete response
CRS: cytokine release syndrome
DCR: disease control rate
DIPG: diffuse intrinsic pontine glioma
DLT: dose-limiting toxicity
DMG: diffuse midline glioma
DLBCL: diffuse large B-cell lymphoma
DNMT3A: DNA methyltransferase 3 alpha
dnTGF-βRII: dominant-negative TGF-β receptor type II
E:T: effector-to-target
ECM: extracellular matrix
FAP: fibroblast activation protein
G/GEJ: gastric/gastroesophageal junction
GMP: Good Manufacturing Practice
GPC3: glypican-3
HDAC: histone deacetylase
HCC: hepatocellular carcinoma
HPA: hypothalamic-pituitary-adrenal
HR: hazard ratio
HRE: hypoxia-response element
iCasp9: inducible caspase 9
ICANS: immune effector cell-associated neurotoxicity syndrome
ICV: intracerebroventricular
IDO: indoleamine 2,3-dioxygenase
IFP: interstitial fluid pressure
IL: interleukin
LOH: loss of heterozygosity
LNP: lipid nanoparticle
MDSC: myeloid-derived suppressor cell
MPC: mitochondrial pyruvate carrier
NFAT: Nuclear Factor of Activated T-cells
NR4A: Nuclear Receptor Subfamily 4 Group A
NSCLC: non-small cell lung cancer
ODD: oxygen-dependent degradation domain
ORR: objective response rate
OS: overall survival
OXPHOS: oxidative phosphorylation
PFS: progression-free survival
PGE2: prostaglandin E2
PKA: protein kinase A
RIAD: regulatory subunit I anchoring disruptor
RMAT: Regenerative Medicine Advanced Therapy
ROS: reactive oxygen species
SCFA: short-chain fatty acid
scFv: single-chain variable fragment
SNIP: Signal Neutralization by an Inhibitable Protease
synNotch: synthetic Notch
TCR: T-cell receptor
TGF-β: transforming growth factor-beta
TIAN: Tumor Inflammation-Associated Neurotoxicity
TIL: tumor-infiltrating lymphocyte
tLNP: targeted lipid nanoparticle
TME: tumor microenvironment
TOX: Thymocyte Selection-Associated High Mobility Group Box
TPC: treatment of physician’s choice
Treg: regulatory T cell
T_SCM: stem cell memory T cell
V2V: vein-to-vein
